# Energy Management and Economic Considerations of Intermittent Photovoltaic-Driven Electrochemical Ammonia Production

**DOI:** 10.1021/acs.energyfuels.3c02123

**Published:** 2023-09-23

**Authors:** Sai A. Varanasi, Carlos A. Fernández, Marta C. Hatzell

**Affiliations:** † George W. Woodruff School of Mechanical Engineering, Georgia Institute of Technology, Atlanta, Georgia 30318, United States; ‡ School of Chemical and Biomolecular Engineering, Georgia Institute of Technology, Atlanta, Georgia 30318, United States

## Abstract

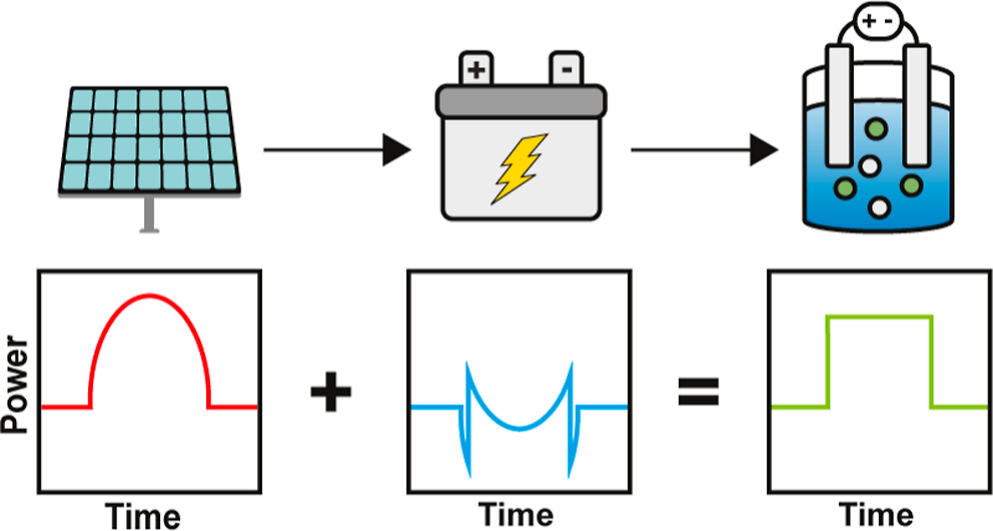

As the energy sector shifts from fossil fuels to renewable energy, there is a need for long-duration energy storage solutions to handle the intermittency of renewable electricity. Electrofuels, or fuels synthesized from excess electricity, are an emerging medium poised to meet long-duration energy storage requirements. Ammonia as an electrofuel is potentially ideal because ammonia has a relatively low liquefaction pressure, indicating that ammonia can be easily stored and transported. Here, we develop a framework to optimize the electrochemical production of ammonia powered by intermittent photovoltaic power. We also explore various buyback policies to understand the impact that policy has on the cost of intermittent ammonia and optimal sizing ratios. The optimal ratio of the photovoltaic to the electrolyzer is ∼3.7 MW_PV_/MW_ELEC_ for a system that is completely powered by renewable photovoltaic power and operates intermittently. The optimal ratio of the photovoltaic to the electrolyzer is ∼3.3 MW_PV_/MW_ELEC_ for a system that uses photovoltaics in conjunction with grid electricity and operates continuously. For the purchase price at the avoided cost of electricity, the optimal ratio of the solar panel to the electrolyzer increases to ∼4 MW_PV_/MW_ELEC_ for a system that can only sell to the grid and ∼5 MW_PV_/MW_ELEC_ for a system that can buy and sell electricity to the grid at the avoided cost. Optimizing energy management by setting auxiliary battery size limits is essential to reducing ammonia costs, and the optimal battery size decreases as the buyback price of electricity increases. Finally, we find that systems connected to the grid and operating continuously have emissions comparable to the Haber–Bosch process because of the current emissions tied to the United States electricity generation. Thus, unless the grid is completely decarbonized, it is essential to create electrofuels that rely minimally on grid electricity.

## Introduction

Around 80% of the ammonia produced through the Haber–Bosch process is used to manufacture synthetic nitrogen-based fertilizers. With the expansion of the global population by more than 25% to 9.9 billion in 2050, ammonia production must increase by up to 80% to meet future projections of fertilizer demand.^1^ This dependence on ammonia to feed the global population highlights the importance of ammonia for society and limits other applications of ammonia, such as the use of ammonia as an energy vector.^2,3^ Furthermore, the considerable increase in food demand has caused an increase in food production and emissions related to food production.^4^

With the emergence of renewable ammonia production, another potential use of ammonia is for long-term energy storage.^5^ There are significant short-term and seasonal differences in the availability of wind and solar energy due to seasonal changes in solar irradiance and wind patterns.^6^ To integrate ammonia production with renewable energy sources, the ammonia production system must be able to operate dynamically to handle intermittency.^7,8^ Batteries are ideal for short-term storage but lack the capacity for long-term storage on the order of several months. Chemical energy storage can store large amounts of energy at low prices compared to other technologies.^9^ Ammonia has special advantages over other chemical fuels, such as hydrogen, because ammonia is cheaper, safer, and easier to store. Ammonia can be liquefied and stored at −33° C at atmospheric pressure or 8 bar at room temperature.^10^ Only 0.1 and 0.6% of the stored energy is needed to liquefy and store ammonia compared to hydrogen, where 44.7 and 2.3% of the energy is used to liquefy and store hydrogen, respectively.^11^ Many predict that ammonia will be an important part of the future energy landscape, serving as a green energy carrier.^12,13^ Ammonia can be converted to electricity with an efficiency of approximately 40–55% depending on the type of engine used.^14^ Although this technology has not been used on a large scale, a study of renewable ammonia production on a small island has shown the potential to implement this technology.^15^

The current process for ammonia production is the Haber–Bosch process. This process is capable of producing ammonia at a very low cost ($200/ton)^16^ using economies of scale with large ammonia plants. The cost of ammonia from the Haber–Bosch process is highly dependent on regional natural gas prices, availability, and transportation costs.^17^ The Haber–Bosch process is dependent on fossil fuel energy (consuming 2% of global fossil fuels).^14,18^ Thus, the use of the Haber–Bosch process to make liquid ammonia as an energy storage medium is unlikely since this would reintroduce carbon emissions into electrification if Haber–Bosch ammonia is used as a medium for energy storage. Furthermore, thermal-based processes also suffer from long startup times, which may make these technologies incompatible with renewable energy. On the other hand, electrochemical processes have the potential to decarbonize ammonia production when coupled with a renewable source of energy and thus are a promising platform technology for generating electrofuels.

On a daily basis, solar energy production peaks in the middle of the day and produces periods without energy production at night.^19^ Intermittency becomes an issue when system components require long startup times. When the photovoltaic system produces no energy, it must stop and then start again. Some components within an electrochemical ammonia plant do not have instantaneous startup times. For example, air separation units require startup times that can range from minutes to 2 h.^20^ This delay in operation decreases energy efficiency and can contribute to increased operational costs. Therefore, with the intermittency of solar energy, there is a potential for wasted energy based on the frequency with which the system is turned off and started.^20^

Previous techno-economic models have shown a completely renewable and flexible electrochemical system^7^ analyzed using mixed integer linear programming and continuous grid-powered ammonia production.^8,16^ These models have demonstrated the potential viability of electrochemical ammonia production as technologies improve beyond the lab scale. This model is unique in that it uses brute-force optimization, which allows for extensive sensitivity analysis by examining the entire variable space. We specifically focus on optimal sizing and understanding which component sizes matter most to the cost of ammonia. Additionally, this model explores the impact of policy on optimal system sizing, which has implications for selecting ideal plant conditions when policies can change over the lifetime of a system.

Here, we model an electrochemical ammonia system that is completely decarbonized and runs on an intermittent basis with solar energy and a system that runs constantly with both solar energy and energy from the grid. We calculate the capital cost of the system and simulate the annual production of the system using annual hourly solar irradiance data. Then, we calculate the production costs per year and use the discount rate to calculate the price of ammonia per ton. We implement various buyback policy considerations to assess the impact that policy considerations may have on the price of ammonia and find ideal size ratios between various components of the system. We also show the impact of proper auxiliary battery sizing and energy management on the price of ammonia. All ammonia in this system is either sold as fertilizer, sold for chemical processes, or sold for energy storage applications. However, we do not directly model the integration and use of ammonia for onsite energy storage.

## Methods

### Objective Function

The objective function minimizes the levelized cost of ammonia by minimizing the sum of all capital and operating costs for the complete system, normalized by the total ammonia produced throughout the operation of the plant. The capital costs are one-time costs and do not vary with the operation of the system as they only depend on system sizing. However, the operation costs and the quantity of ammonia produced are calculated by adding the values for each time interval.
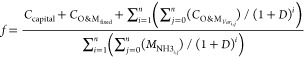
1where *C*_capital_ is the total capital cost of the ammonia production system,  is the fixed operation and maintenance cost of the system,  is the variable operation and maintenance cost of the system, *D* is the discount rate, *i* is the sum corresponding to each year of operation, and *j* corresponds to the sum for each time interval (i.e., Δ*j* = 60 min). The variable operation and maintenance costs depend on the amount of power purchased or sold to the grid at any given time.

### System-Level Constraints

At a system level, we set energy and mass balance constraints that must be met in order for the system to be considered viable. The energy balance constraint ensures that energy generation and consumption are balanced at each time step.
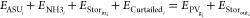
2where the terms on the left of the equality represent the energy consumed by the air separation unit, the ammonia generation system, the energy going into the battery, and the PV energy curtailed at any time interval. The terms toward the right of equality represent the energy generated by the PV system and the energy leaving the battery at any given time. These quantities must be equal at any period of time. Additionally, if the energy leaving the battery at any point in time is greater than the energy stored in the battery before the beginning of the time step, the system will not meet the energy constraint.

The mass balance constraints that must be met in order for the system to be valid are related to the nitrogen produced and consumed at any time step.
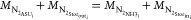
3where the terms on the left of the equality represent the nitrogen generated by the air separation unit, and the nitrogen leaving the storage tank at any period of time. The terms toward the right of equality represent the nitrogen consumed by the ammonia generation system and the nitrogen entering the storage tank at any given time. Additionally, for every time step, the nitrogen stored must be positive.

### System Description

The location of the modeled system is the Solana Generating Plant (32.9, −113) in Arizona due to the high potential for solar energy in the southwest of the United States and the existing solar infrastructure in that location. The hourly solar irradiance for a year was found using PVWatts for that location. The land cost used is a low estimate of the land cost in Arizona, $1000/hectare,^21^ and the commercial cost of electricity in AZ is $0.11/kW h.^22^

For the intermittent system, all energy is supplied by the photovoltaic system. The years’ worth of hourly solar irradiance data is available at the plant location using the PVWatts tool.^23^ The energy produced by the solar panel per hour can be calculated by
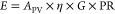
4where *A*_PV_ is the area of the solar panel, η is the efficiency of the solar panel, *G* is the solar irradiance for each hour, and PR is the cell performance ratio, estimated at 0.75 to account for constant losses in the system due to shadows, DC to AC conversion, and temperature.^24^

The cost of the solar panel is calculated with the NREL PV LCOE calculator for a solar panel with 19% efficiency and single-axis tracking.^25^ The excess energy from the solar panels is directed to a lithium-ion battery in the intermittent system. The battery is oversized by 20% due to the inability to completely discharge the battery.^26^

The ammonia electrolyzer unit is modeled as a reactor with nitrogen and hydrogen with 30% faradaic efficiency.^16^ The air separation unit is modeled as a pressure swing adsorption (PSA) unit with 99.99% purity since PSA units are economical for a wide range of flow rates and have a small startup time^20,27^ We assume that the lifetime of the electrolyzers and solar panels is 25 years.^25^ This is slightly less than previous lifetime estimates of 30 years for electrolyzer systems,^16,28^ but it allows the electrolyzer lifetime and solar panel lifetime to be comparable. Due to discount rates, the last few years of production do not generate much income, making this assumption have a relatively low impact on the model. During that time, the only system that must be replaced is the battery, which is in the middle of its useful life.^26^ The energy requirements for the components are shown in Table 1.

**Table 1:** Energy Requirement for the Air Separation Unit and Hydrogen Electrolyzer

component	ASU^29^	electrolyzer^16^
energy requirement	0.0164 MW/ton/day	0.437 MW/ton/day

The systems studied here have not been proven at commercially relevant scales. However, we hope that the methods and findings in this paper might help highlight the importance of system sizing and operation when designing flexible electrochemical systems that integrate with renewable energy sources.

### System Operation

#### Intermittent System Operation

**Figure 1:**
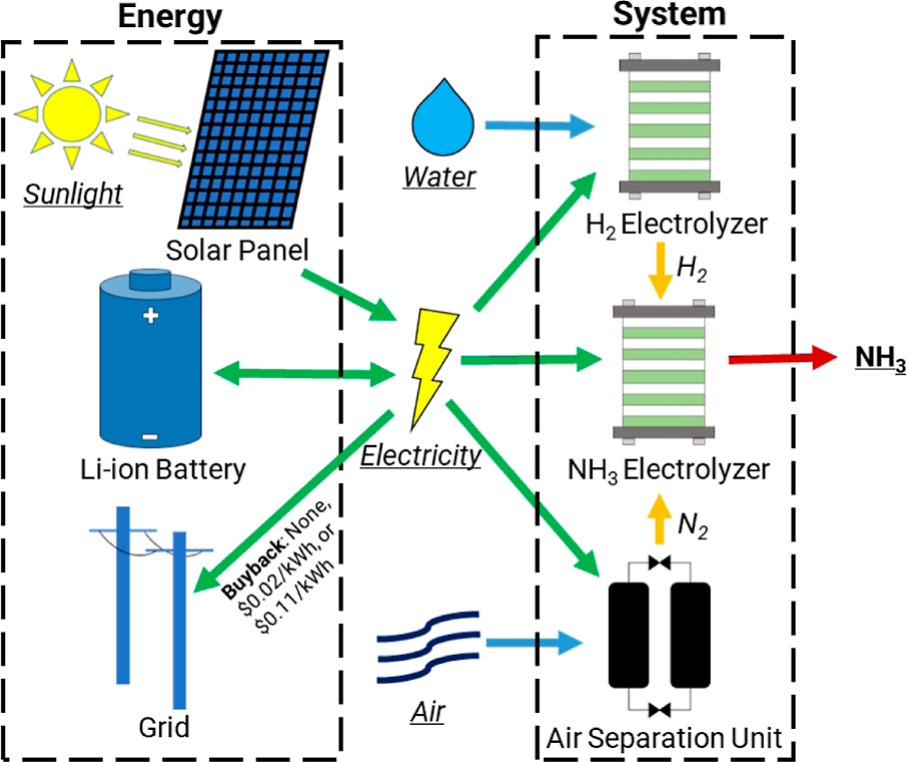
Flow diagram of an intermittent system powered by a solar photovoltaic panel and battery for ammonia production. This system is only able to sell curtailed energy to the grid and it consists of an air separation unit, a water electrolyzer, and an ammonia electrolyzer.

The system operates intermittently, with changes in operation occurring at each hour. The two components of the system, the hydrogen and ammonia electrolyzers, and the air separation unit, can be in on-stage or off-stage conditions (Figure 1). To model the startup time, when the air separation unit turns on, there is a 30 min window where the unit is on, but no nitrogen production occurs because the nitrogen output does not meet the purity required by electrochemical systems.
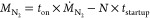
5where  is the total mass of nitrogen produced by the air separation unit in a year, *t*_on_ is the amount of time that the air separation unit is running during the year,  is the nitrogen flow rate from the air separation unit when it is turned on, *N* is the number of times the air separation unit must start again, and *t*_startup_ is the startup time needed for the PSA air separation unit (i.e., 30 min).^20^

The electrolyzer unit can be turned on immediately. The system makes a decision on whether to turn on and off each component of the system based on how much energy is available at a given time. The energy available at a given time is calculated based on the amount of energy available from the solar panel during that hour and the amount of energy available in the battery at that time
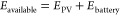
6

If enough energy is available for the ASU to run, that is prioritized to avoid loss of energy due to its startup time. Then, if enough energy is available for the electrolyzer to run, it runs as well. The energy is first used from the photovoltaic system and then taken from the battery. If there is an excess of energy and the energy is below the battery size limit, then the battery is charged. If the energy level is above the battery size limit, the energy is curtailed—either released as heat or sold back to the grid for a buyback cost. The size of the battery is constrained by an input value, the battery size factor, which is multiplied by the total power requirement of the system.

#### Constant System Operation

**Figure 2:**
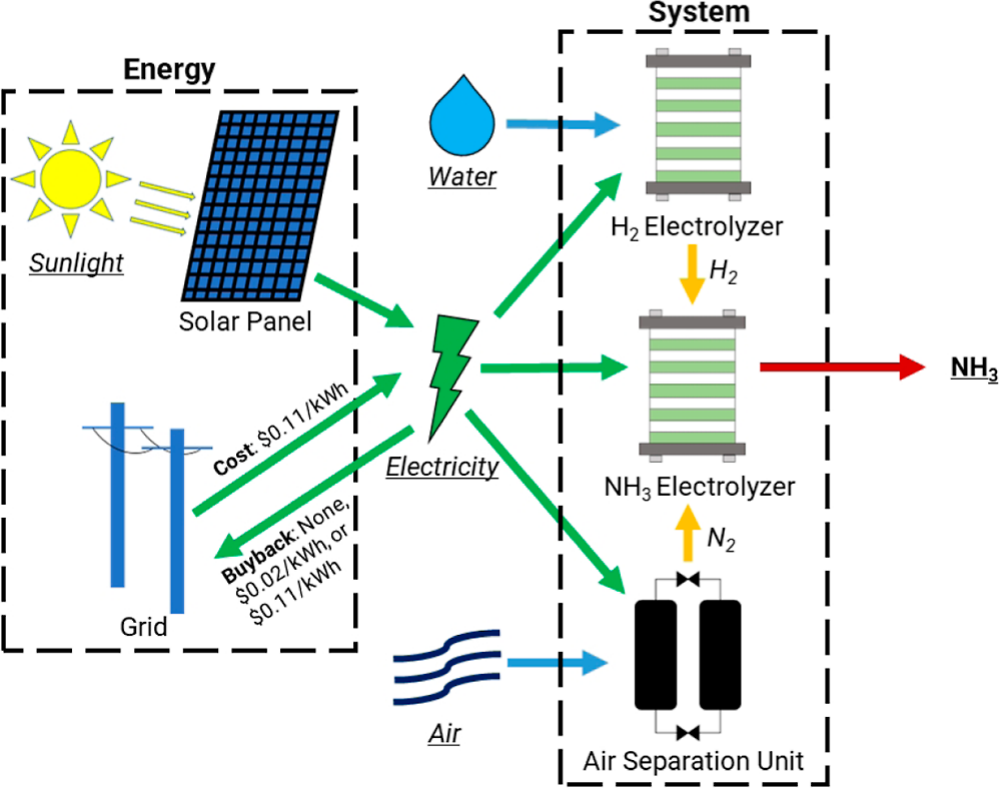
Flow diagram of a continuous system powered by a solar photovoltaic system and fully connected to the grid. This system consists of an air separation unit, a water electrolyzer, and an ammonia electrolyzer.

In constant system operation (Figure 2), the ammonia system continuously operates at full capacity with energy from the solar panel and the grid. Any extra energy generated by the solar panel in a given hour is sold to the grid or wasted as heat, according to the buyback policy. Any energy needed to keep the system operating continuously is purchased from the grid. The system never turns off, so startup times do not need to be taken into consideration.

For both systems, the yearly degradation is modeled to be 0.7%.^25,30^ This degradation is based on solar panel degradation as that determines the total amount of available energy for ammonia production. Total production over the 25 year lifetime of the system is discounted to account for the degradation of the system.
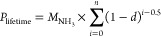
7where *P* is the lifetime ammonia production of the system,  is the annual ammonia production of the system, *n* is the system lifetime (i.e., 25 years), and d is the degradation rate of the system.

To calculate the excess carbon dioxide emitted by this system compared to the completely photovoltaic-driven system, the amount of energy obtained from the grid is used. We do not consider the offset carbon emissions of energy sold to the grid due to the small impact it makes in comparison to the energy in the grid.
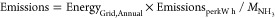
8where Energy_Grid,Annual_ is the annual energy taken from the grid, Emissions_perkWh_ is the amount of carbon dioxide emitted per kWh of energy from the grid, 0.385 ,^31^ and  is the annual production of ammonia in tons.

### Cost Modeling

There are several cost modeling values used in this section, as defined in Table 2.

**Table 2:** Economic Parameters for a Photovoltaic Array Capital Cost, Land Cost, and Battery Capital Cost

variable	*C*_PV,area_(25)	*C*_PV,power_(25)	*C*_land_(21)	*C*_battery,energy_(26)	*C*_battery,power_(26)
value	108.67 $/m^2^	200 $/kW	640 $/ha	285 $/kWh	306 $/kW

The cost of the water electrolyzer is 600 $/kW.^32,33^ The capital cost estimate for water electrolysis comes from literature values that have analyzed materials and performance improvements and created future projections for the installed capital cost of PEM water electrolyzers. The cost of the ammonia electrolyzer and the balance of plant costs are taken from previous work and are detailed in the Supporting Information.^16^

The cost of the PSA ASU is^27^
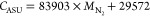
9

The cost of the PV system can be found as follows^21,25^
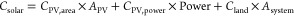
10where the area of the system is the area of the solar panel over the ground coverage ratio of 0.6
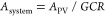
11

which accounts for the assumption that the area of the solar panel is 40% of the land area.

The cost of the battery is^26^
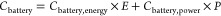
12where *P* is the maximum power the battery can deliver in kW, and *E* is the amount of energy in the battery in kW h.

The cost of N_2_ storage is considered negligible. The operating and maintenance cost of the electrolyzer system, ASU, and battery is 2% of the annual capital cost.^20^ The operation and maintenance cost of solar panels is $17.46 per kW.^25^ Energy buyback is a policy that can decrease the cost of ammonia by allowing the plant to sell excess solar energy produced and not stored by the battery. Energy buyback policies can allow energy to be purchased back at the retail price or at the avoided cost, which is the cost of producing electricity for the energy company.^34^ The energy buyback policy can be set up in three different ways. The buyback cost can either be $0.00 when energy is wasted, $0.02/kW h in avoided costing, or $0.11/kW h in retail costing. Any energy bought from the grid in the constant system is bought at a retail cost of $0.11/kW h.^22^

The levelized cost of ammonia (LCOA) is calculated by totaling the capital cost of the solar panels, battery, electrolyzer, and ASU and adding the annual cost discounted each year.
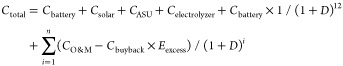
13where *n* is the system lifetime of 25 years, and *D* is the discount rate of 6.3%. During year 12, the battery is replaced to account for its shorter lifetime. The LCOA can be found by
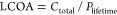
14

## Results

### Islanded Photovoltaic-Driven Ammonia Production

#### Optimizing Component Sizing

In order to model an ammonia production system that operates intermittently, we considered a photovoltaic-driven system connected to the grid. To ensure carbon-free ammonia production, this system is only able to sell electricity to the grid. However, all the electricity used to produce ammonia must come from the photovoltaic system (Figure 1). We analyze three scenarios for grid interconnection. The first scenario models a completely islanded system in which none of the curtailed electricity is sold back to the grid. The second scenario considers a connected grid system in which the buyback rate is equal to the avoided cost of the grid (0.02 $/kW h). Finally, the third scenario considers a grid-connected system in which the buyback rate is equal to the retail cost of electricity (0.11 $/kW h). For each scenario, we optimized the photovoltaic size, the battery size factor, and the air separation unit size at electrolyzer flow rates between 0 tons/day and 3000 tons/day using a brute-force optimization algorithm. Brute-force optimization works by attempting every possible solution and choosing the one with the lowest cost among all solutions. Because every solution is considered, brute-force optimization ensures convergence to the global minimum. Additionally, it has the benefit of being able to analyze and understand how the system behaves outside of the optimal solution. The optimal linear size ratios were analyzed to understand the optimal system size for each component when compared to the electrolyzer. These ratios can be applied to larger systems, but this is a realistic upper limit for electrochemical systems. We examined the effect of each independent variable on the ammonia production cost, holding the other two variables fixed at their optimal values calculated by the brute-force optimization algorithm. For air separation specifically, the ratio was adjusted higher than the optimal calculation to ensure that sufficient nitrogen is produced with downtimes that are longer than those considered with the solar input curve. When doing sweeps of each variable for plotting to show ranges of operating with low costs, the other two variables were fixed at their optimal values as calculated with brute-force optimization. For air separation specifically, the ratio was adjusted slightly higher than the optimal calculated to ensure that sufficient nitrogen production occurs at all flow rates.

**Figure 3:**
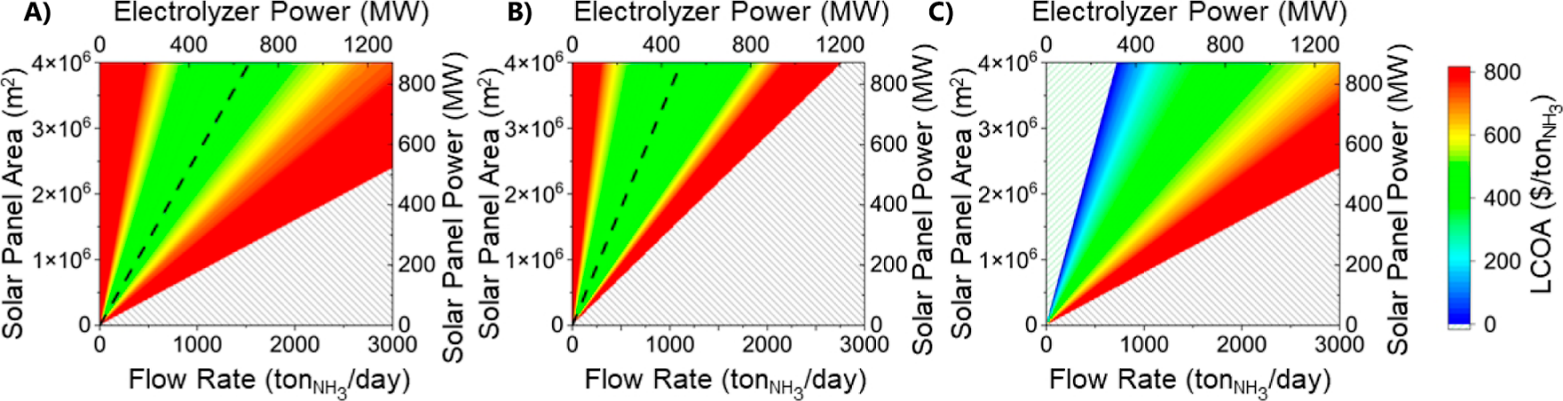
LCOA for a range of PV areas and electrolyzer flow rates for buyback costs of (A) $0.00/kW h, (B) $0.02/kW h, and (C) $0.11/kW h with optimal ratios between the two shown.

For the first scenario, which models an islanded system with a purchase of electricity at a cost of 0.00 $/kWh, the optimal ratio between the electrolyzer size and the PV system capacity is 3300 m^2^/(tons/day) shown by the black dashed line (Figure 3a). This is equivalent to a ratio of electrolyzer power to photovoltaic power of 3.72 MW_PV_/MW_NH3_. For this scenario, the minimum levelized cost achieved for ammonia is 416 $/ton_NH3_ for an electrolyzer flow rate of 1530 tons_NH3_/day and a PV area of 398.4 ha. For a family farm of 100 hectares (which requires 0.03 tons/day of ammonia), the optimal photovoltaic-driven ammonia production system would occupy 0.01 hectares, which is equivalent to 0.01% of the arable land. This indicates that the land used by an islanded PV-driven electrochemical ammonia production system will not interfere with the land allocated for agriculture.

For the second scenario, which assumed buyback at the avoided cost of electricity (0.02 $/kWh), the ratio grows to 3600 m^2^/(tons/day) since a larger photovoltaic produces more energy that can be sold to offset the cost of ammonia (Figure 3b). The optimal ratio is equivalent to a ratio of electrolyzer power to photovoltaic power of 4.06 MW_PV_/MW_NH3_. Thus, the minimum ammonia production cost decreases to 392 $/ton_NH3_ for an electrolyzer flow rate of 1108 tons_NH3_/day and a PV area of 397 ha. There is only a marginal increase in land use to 0.011%.

Finally, for the third scenario (Figure 3c), which assumed buyback at a retail price of electricity (0.11 $/kW h). The optimal case is to produce only solar energy and not ammonia since the levelized cost of energy is approximately $ 0.04/kW h,^25^ meaning that all excess energy is sold for profit. However, the break-even point in which the costs to produce ammonia are completely offset by the profits from the electricity sold is achieved with a system with a ratio of 5600 m^2^/(tons/day) or 6.32 MW_PV_/MW_NH3_. There are policy challenges with this scenario. Several states have implemented policies that limit the capacity of systems that can sell electricity at retail prices.^35^ Therefore, most commercial and residential photovoltaic systems sell their electricity at a reduced cost. Furthermore, since the land allocated for these systems will likely compete with the land used for agriculture, it is important to also minimize the footprint of the land.

**Figure 4:**
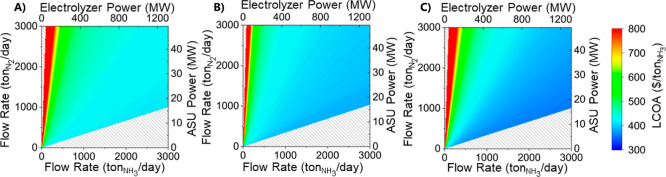
LCOA for a range of nitrogen and electrolyzer flow rates for buyback costs of (A) $0.00/kW h, (B) $0.02/kW h, and (C) $0.11/kW h with optimal ratios between the two shown.

For sizing the air separation unit, the ammonia production cost is plotted across ranges of both nitrogen flow rates for the air separation unit and ammonia flow rates for the electrolyzer. The optimal ratio for all cases is to have the minimum possible air separation unit size as enough nitrogen is generated to produce the given amount of ammonia. The smaller the ASU, the lower the capital cost, leading to a lower LCOA. Therefore, it is best for the ASU to be as small as possible (Figure 4). The case where the ASU is undersized and cannot produce enough nitrogen to keep up with the electrolyzer demand is demonstrated by the hash marks in Figure 4. Therefore, the best ratios occur with the smallest ASUs that produce exactly enough nitrogen for the ammonia produced, which is about 0.35 ton N_2_/ton NH3_3_ or 0.013 MW/MW.

#### Energy Management

The most important factor in energy management in the scenarios we tested was efficient battery size. Allowing the battery to be as large as needed to conserve all the solar energy possible is more expensive than controlling the battery size and allowing some energy to be wasted.

**Figure 5:**
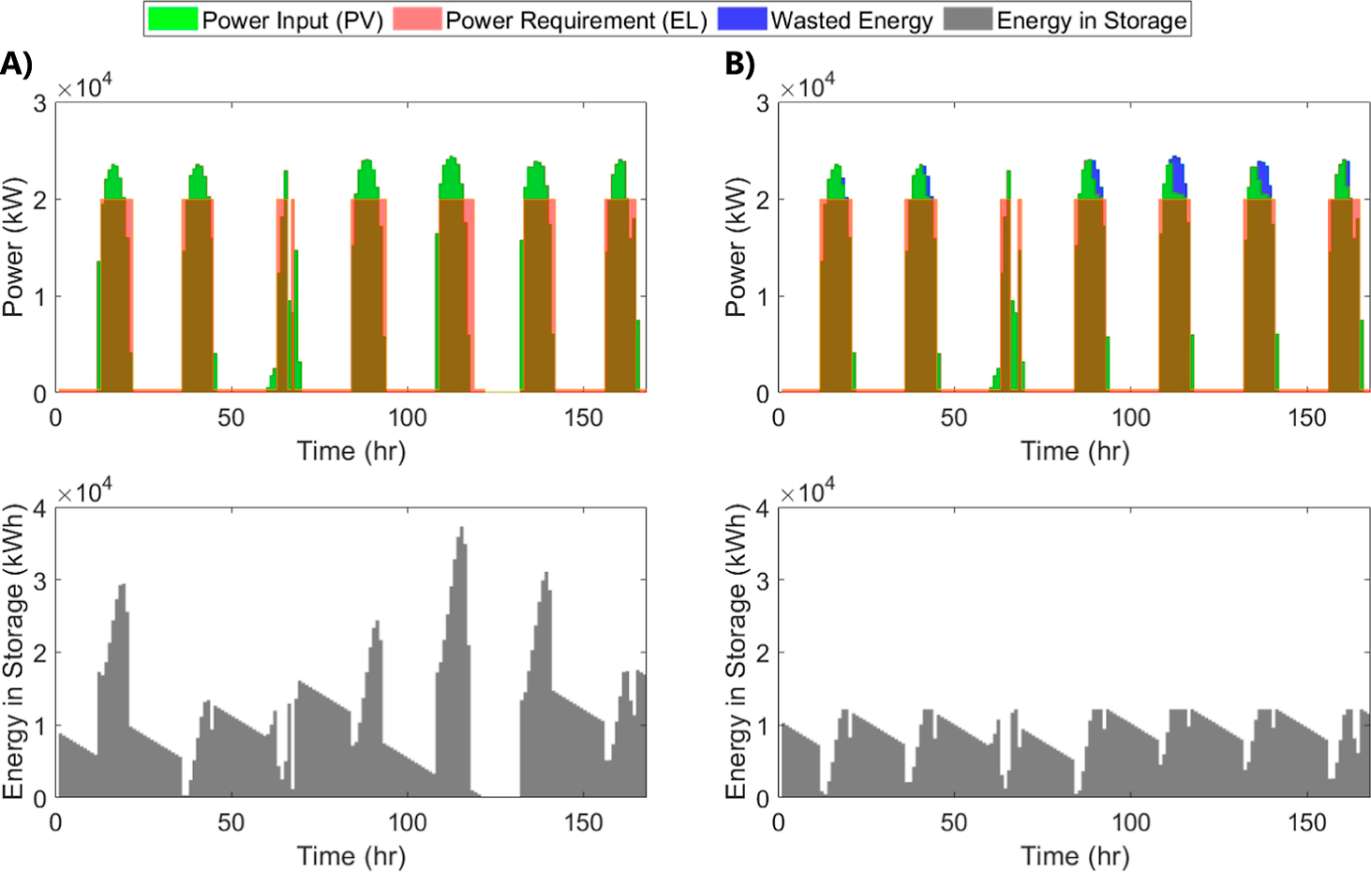
Power requirements, inputs, and energy storage needs for a week of operation in cases (A) no energy management and (B) battery size limitations at the estimated best ratio.

In the case where there is no size constraint on the battery (Figure 5a), the maximum energy in storage is almost three times the maximum energy in storage with a size limitation (Figure 5b). Therefore, the capital cost of the battery increases by three times in the first case without much change in ammonia production. Therefore, managing energy and allowing some energy to be wasted or sold to the grid is more profitable than using all the energy produced by the solar photovoltaic system.

The most important factor in ensuring efficient energy management in the system is the maximum battery size. If there is no limit to the battery size, all the excess solar energy will be conserved and used to produce ammonia. This will lead to more ammonia production. However, the additional capital cost required to purchase a larger battery will result in larger ammonia production costs. Hence, limiting the battery size allows some of the excess energy to be used to produce ammonia while still maintaining low ammonia production costs.

In order to limit the battery size in a comparable way between system flow rates, we introduce a normalized variable for battery size, which we define as the battery size factor. This factor is the maximum battery size constraint in kWh divided by the total system power requirement in kW. This factor allows systems with small and large flow rates to be compared and optimized in a similar manner.

**Figure 6:**
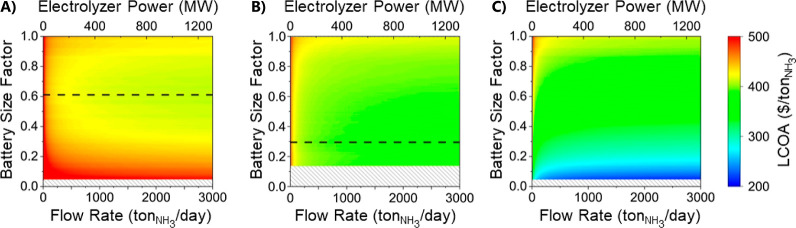
LCOA for a range of battery size limits and electrolyzer flow rates for buyback costs of (A) $0.00/kW h, (B) $0.02/kW h, and (C) $0.11/kW h with optimal ratios between the two shown. The dashed line shows the optimal battery size for the smallest LCOA.

For battery sizing, the LCOA is plotted for various electrolyzer flow rates and battery size factors. For the completely islanded system (Figure 6a), the optimal battery size factor is 0.61. This size of the battery allows sufficient energy storage to produce ammonia efficiently without substantially increasing the capital cost of the system so that additional ammonia production is no longer profitable.

For the avoidance policy (Figure 6b), the best battery size factor is 0.2. This is due to the optimal case having a larger photovoltaic, which leads to more energy being available to the system on demand. Additionally, saving energy for later with the high capital cost of the battery makes the additional ammonia produced when excess energy is not economical.

For the retail buyback policy (Figure 6c), the best battery size factor would be close to 0. The highest profits will occur when the battery does not exist and all the energy is sold to the grid, as the levelized cost of energy is less than the retail cost of energy. In an optimal version of this system, no ammonia would be produced. However, producing ammonia with a photovoltaic system and a small battery and selling the rest of the energy would yield a significantly smaller LCOA in this policy scenario than in the avoidance one. Therefore, making the choice to have as large a photovoltaic in this case would drive the cost of ammonia down. However, in other locations where the cost of solar energy is higher due to less solar irradiance, this scenario may converge to an optimal scenario that involves producing ammonia.

Limiting the size of the battery is an efficient method of effectively managing energy. When the battery size is left to accommodate the amount of energy that could be stored to prioritize not wasting any energy, the battery becomes very large, leading to very high capital costs. However, since the battery only reaches full capacity in a few days, there is little return on investment for a larger battery. Finding the best limit for battery size allows the entire battery capacity to be used efficiently.

**Figure 7:**
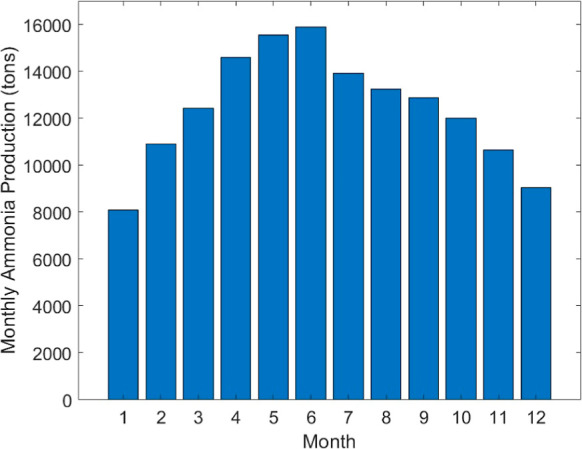
Monthly production of ammonia for the first year of operation for 12 equally spaced months.

For the intermittent system, the production varies monthly, but energy is managed, so the battery goes through charging and discharging cycles on a daily or weekly basis. Throughout the year, different amounts of ammonia are produced, rather than the battery storing energy in the summer to use in the winter. Splitting up the year into twelve evenly spaced months, the monthly production is shown in Figure 7. The system produces more ammonia during the summer when solar energy is more abundant and less ammonia during the winter when solar energy is less abundant. This monthly change could be beneficial for fertilizers due to the increase in demand for fertilizers during the spring and summer months. Additionally, for long-term energy storage, the excess ammonia produced in the summer could be converted into energy during the winter, when solar energy is less available.

Comparing the intermittent system to other proposed systems, the LCOA is quite low (400 $/ton). Others report fully renewable electrochemical ammonia to cost around 900–1000 $/ton^7^ and a grid-connected system to cost around 540–640 $/ton.^8^ For all systems, the biggest contributor to cost is the capital cost of the PV solar installation. The lower costs in our results can be explained because our PV capital costs and electrolyzer capital costs align with those of the lower capital cost estimates of utility-scale systems in 2050. However, a similar behavior of oversizing the renewable energy source and minimizing storage to achieve the best performance was seen in our paper and previous literature.

### Grid-Connected PV-Driven Ammonia Production

In the constant system, the ASU is sized stoichiometrically for the electrolyzer flow rate. The battery is not part of the system and does not need to be sized as energy flows into and out of the grid as needed. Therefore, the only sizing consideration is the ratio between the solar panel area and the electrolyzer flow rate. Here, we have used a fixed retail electricity purchase price and varied the possible buyback costs depending on different policy scenarios. We did this to understand the effect of buyback policies rather than to optimize a system with transient electricity prices.

**Figure 8:**
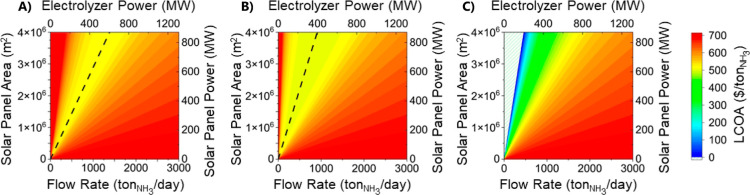
LCOA for a range of PV areas and electrolyzer flow rates for buyback costs of (A) $0.00/kW h, (B) $0.02/kW h, and (C) $0.11/kW h with optimal ratios between the two shown.

For a constant system where there is no buyback policy, the optimal ratio between the photovoltaic area and the electrolyzer flow rate is 2900 m^2^/(tons/day) shown by the dashed black line (Figure 8a). This is equivalent to a ratio of PV power to electrolyzer power of 3.27 MW_PV_/MW_NH3_. The smallest ammonia production cost is 514 $/ton_NH3_ for an electrolyzer flow rate of 1377 tons_NH3_/day and a PV area of 400 ha. This is a lower ratio than that of the intermittent system, which means that for this scenario, the photovoltaic is lower. This is because all excess photovoltaic energy is wasted and not stored in a battery, and there is no scarcity of energy since the system is connected to the grid. Therefore, the solar panel can be smaller since not all the energy is needed, and any excess energy is wasted.

For the avoidance buyback policy (Figure 8b), the optimal ratio is 4400 m^2^/(tons/day), equivalent to the ratio of photovoltaic power to electrolyzer power of 4.96 MW_PV_/MW_NH3_. In this scenario, the lowest ammonia production cost is 499 $/ton_NH3_ for an electrolyzer flow rate of 895.8 tons_NH3_/day and a PV area of 400 ha. This ratio grows quickly as the added benefit of selling energy to the grid helps offset the cost of ammonia, and the difference in cost of energy is very large between the grid and solar panels. As a result of the low cost of solar energy, the size of the solar panel should be larger to allow more of the used energy to come from solar energy. Since excess energy helps offset the capital cost of the solar panel, a larger solar panel is more optimal.

For the retail buyback scenario (Figure 8c), the optimal case would be to have the solar panel operating as large as possible with no ammonia production. There is a considerable portion of the plot in green hashed lines (Figure 8c) where the PV area is so large and the electrolyzer flow rate is so small that the LCOA is negative since the capital costs of the entire system are offset by the profit that selling solar energy makes. This is due to the low cost of solar energy and the higher retail cost. Therefore, large profits can be made by selling solar energy.

The LCOA of ammonia is larger in this ideally-sized constant system than in the ideally-sized intermittent system. This is due to the lack of economies of scale for an electrochemical system. Additionally, because of the low startup time of all components of the system, the advantage of cheap solar energy allows the intermittent system to produce ammonia at a lower cost. This shows the advantages of cheap solar energy and intermittent operation over the potential advantage of constant operation in a higher-capital-cost system.

**Figure 9:**
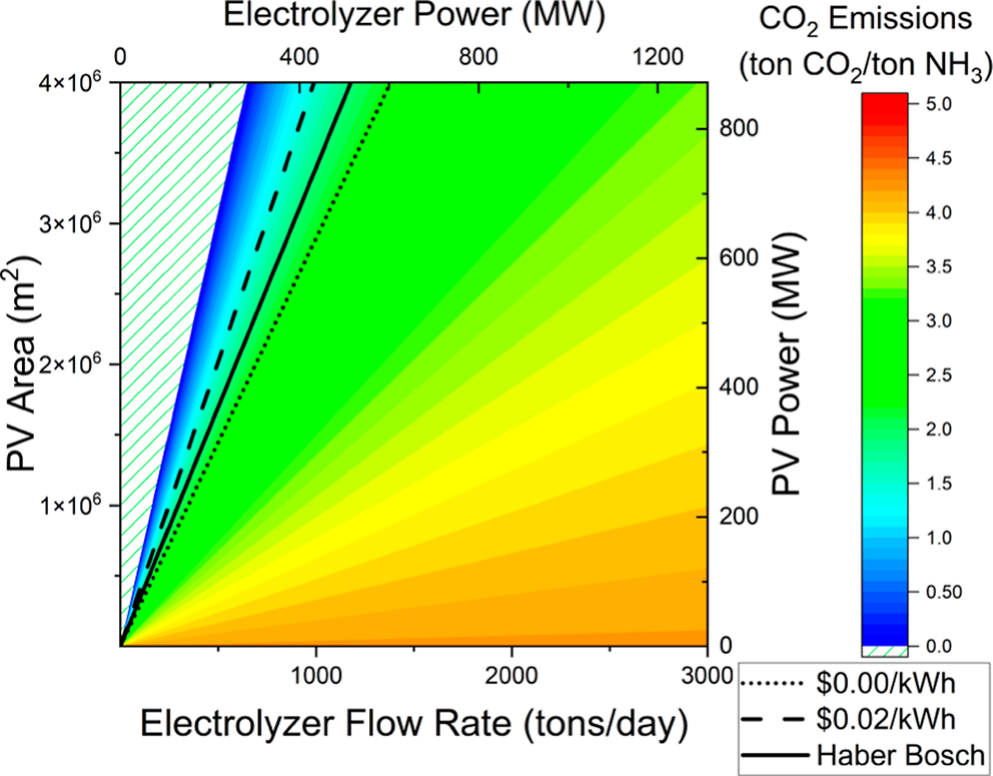
Carbon dioxide emissions for a range of PV areas and electrolyzer flow rates with minimized cost ratios shown for no buyback, avoidance buyback, and the Haber–Bosch process.

In a constant system, cost is not the only factor that needs to be considered. There are extra carbon dioxide emissions that are not present when the intermittent system is used, as all energy is renewable in that case. The carbon dioxide emissions per ton of ammonia produced are the smallest when the electrolyzer flow rate is smaller and the solar panel area is larger (Figure 9). In the best case, the Haber–Bosch process releases 1.9 ton_CO2_/ton_NH3_ during ammonia production.^36^ When offsetting the carbon emissions for energy going back to the grid, in the case without a monetary buyback incentive (Figure 9, line A), the carbon dioxide emissions are higher per ton of ammonia than in the Haber–Bosch process (Figure 9, line HB). However, when a 0.02 $/kWh buyback policy is implemented, there are fewer carbon dioxide emissions than for Haber–Bosch for the optimal LCOA ratio (Figure 9, line B). This illustrates the importance of the buyback policy in shifting the optimal solar panel to electrolyzer size ratio in order to reduce overall carbon emissions.

## Conclusions

Here, we analyzed the potential of a continuously operated, fully photovoltaic-driven, islanded, intermittent electrochemical ammonia production plant. We also examined a partially photovoltaic-driven electrochemical ammonia plant. We find that to minimize LCOA, sizing systems based on the ratios between the air separation unit, the electrolyzer, and the photovoltaic are crucial. Additionally, for an intermittent system, limiting energy storage is more cost-effective than storing all the energy from the photovoltaic. We find the ideal battery size limit for the system with different energy buyback policies: none, avoidance, and retail. These size ratios and energy management techniques are important considerations when understanding how a buyback policy could reduce the cost of ammonia production. We find that an intermittent system has a smaller LCOA over the lifetime of the system than a constant system; however, this system would also have a higher capital cost. However, over time, the advantages of cheaper solar energy and the reduction of carbon dioxide emissions show promise for intermittent electrochemical ammonia systems.

## Supporting Information Available

System models and model parameters (PDF)
