# Supplementary material for: Energy Management and Economic Considerations of Intermittent Photovoltaic-Driven Electrochemical Ammonia Production
Source: Energy Fuels. 2023 Sep 23;37(19):15222–30. doi: 10.1021/acs.energyfuels.3c02123 (PMC10561136; doi:10.1021/acs.energyfuels.3c02123)
Supplement: Supplementary file 1 — ef3c02123_si_001.pdf [file ef3c02123_si_001.pdf]

Supporting Information: Energy Management and Economic Considerations of Intermittent PV-Driven Electrochemical Ammonia Production

Sai A. Varanasi,[†] Carlos A. Fernández,[†] and Marta C. Hatzell[*,†]

†*George W. Woodruff School of Mechanical Engineering, Georgia Institute of Technology, Atlanta, GA 30318*

‡*School of Chemical and Biomolecular Engineering, Georgia Institute of Technology, Atlanta, GA 30318*

E-mail: marta.hatzell@me.gatech.edu

Variables and Abbreviations

Variable	Symbol	Assumed Value	Units
A_{PV}	Area of solar panel		ha
A_{system}	Total area of system		ha
ASU	Air separation unit		
C_{ASU}	Total cost of ASU		$
$C_{battery}$	Total cost of battery		$
$C_{battery,energy}$	Energy cost of battery[1]	285	$/kWh
$C_{battery,power}$	Power cost of battery[1]	306	$/kW
$C_{capital}$	Total system capital cost		$
$C_{electrolyzer}$	Electrolyzer capital cost		$
C_{land}	Land cost[2]	640	$/ha
$C_{O\&M_{fixed}}$	Fixed operation and maintenance costs		$/year
$C_{O\&M_{variable}}$	Variable operation and maintenance costs		$/year
$C_{PV,area}$	Area cost of PV system[3]	108.67	$/$m^2$
$C_{PV,power}$	Power cost of PV system[3]	200	$/kW
C_{solar}	Total cost of PV system		$
D	Discount rate	6.3	%
E_{ASU_i}	Energy consumed by air separation unit		kWh
$E_{available}$	Energy available to system		kWh
$E_{battery}$	Energy stored in battery		kWh
E_{PV}	Energy generated by PV system		kWh
$E_{PV_{gi}}$	Energy generated by PV system		kWh
$E_{Stor_{in_i}}$	Energy going into battery		kWh
$E_{Stor_{out_i}}$	Energy leaving battery		kWh
$Emissions$	CO_2 emissions generated		tons

$Emissions_{perkWh}$	CO_2 emissions per kWh of energy		tons/kWh
$Energy_{Grid,Annual}$	Energy used from the grid annually		kWh
F	Faraday's Constant	96,485	C/mol
G	Solar irradiance		kW/ha
GCR	Ground coverage ratio	0.6	
i	Current density		A/m^2
i_0	Exchange current density		A/m^2
i_lim	Limiting current density		A/m^2
j	Time interval	1	hour
$LCOA$	Levelized cost of ammonia		$/ton
$\dot{M}_{N_2}$	Nitrogen flow rate		tons/hr
M_{N2}	Nitrogen produced by ASU		tons
$M_{N_{2_{ASU_i}}}$	Nitrogen produced by ASU		tons
$M_{N_{2_{NH3_i}}}$	Nitrogen going to ammonia generation unit		tons
$M_{N_{2_{Stor_{in_i}}}}$	Nitrogen going into storage		tons
$M_{N_{2_{Stor_{out_i}}}}$	Nitrogen taken out of storage		tons
M_{NH_3}	Annual ammonia production		tons/year
N	Number of times ASU is turned on		
n	System lifetime[3]	25	years
$P_lifetime$	Lifetime system ammonia production		tons
PR	Cell performance ratio[4]	75	%
PV	Photovoltaic		
R	Universal gas constant	8.314	$Jmol^{-1}K^{-1}$
$R_electrolyte$	Area specific electrolyte resistance		Ω*m^2
T	Temperature of reaction		K
t_{on}	Time system is on during a year		hours
$t_{startup}$	Startup time required[5]	0.5	hours

V_0	Minimum voltage		V
α	Electron transfer coefficient	0.5	
ΔG	Gibb's free energy		kJ
ΔH	Enthalpy of reaction		kJ
ΔS	Entropy of reaction		kJ / K
η	Solar panel efficiency[3]	19	%
η_{act}	Activation overpotential		V
η_{conc}	Concentration overpotential		V
η_{ohm}	Ohmic overpotential		V
η_{total}	Sum of all overpotentials		V

Electrochemical Ammonia Reactor Modeling

The electrochemical model for the ammonia production system was based on previous work.[6]

The thermodynamic minimum energy required for an electrochemical reaction corresponds to the change in enthalpy of the reaction (ΔH), which can be calculated from the change in the Gibbs free energy (ΔG) and the entropy of the system ($T\Delta S$).

$$\Delta H = \Delta G + T\Delta S \tag{1}$$

Similarly, the thermodynamic minimum voltage for an electrochemical reactor (V_0) is calculated using Faraday's law (where n = 6 and F = 96,485 C/mol) and the Nernst equation.

$$V_0 = \frac{\Delta G}{nF} + \frac{RT}{nF} ln(\frac{a_{reactants}}{a_{products}}) \tag{2}$$

where a_i corresponds to the activity of the reactants and products. Since the reactants and products are gases, their activity is approximated using partial pressures. The activity of water is approximated to be one (a_{H_2O} = 1) because water is the predominant solvent in the solution.

The realistic cell potential (V) is determined by the thermodynamic minimum voltage (V_0) and the sum of all the overpotentials (η_{total}).

$$V = V_0 + \eta_{total} \tag{3}$$

The activation overpotential is calculated using the Butler-Volmer kinetics with an assumed symmetric electron transfer coefficient (α = 0.5).

$$\eta_{act} = \frac{RT}{n\alpha F} * sinh^{-1}(\frac{i}{2i_0}) \tag{4}$$

where R is the universal gas constant, F is the Faraday's constant, T is the reactor temperature, n = 6 for NRR, n = 4 for OER, and n = 2 for HER, i is the operational current and i_0 is the exchange current density.

The ohmic overpotential is described by the Ohm's law

$$\eta_{ohm} = i * R_{electrolyte} \tag{5}$$

where $R_{electrolyte}$ is the area specific resistance of the electrolyte.

The concentration overpotential accounts for the losses due to mass transport.

$$\eta_{conc} = -\frac{RT}{nF} * log(1 - \frac{i_{density}}{i_{lim}}) \tag{6}$$

where the limiting current density (i_{lim}) is the maximum current achievable with the mass transport properties of system.

A more detailed explanation of these calculations can be found on previous work[6]

Capital Cost Estimation

The capital cost includes the cost of main components, peripheral parts, and the balance of plant cost (BoP). The capital cost of the electrolysis cell is based on prices at large production quantities normalized by the surface area of the electrochemical reactor (Table S1).

Table S1: Electrolysis Cell Component Cost

**Parameter**	**Price per Area**	**Source**
Proton Exchange Membrane	\$50/m^2	[7]
Electrode	\$96/m^2	[7]
Bipolar Plate	\$35/m^2	[7]
Peripheral Parts	\$3.46/m^2	[7]
Catalyst (Pt)	\$32,000/kg	
Catalyst Loading	1 g/m^2	

The balance of plant represents all the components that are essential to the function of the system but do not participate in the reaction (pumps, compressors, heat exchangers, storage tanks, electronic components, etc. – Table S2).

Table S2: Balance of Plant Capital Cost[8]

**Parameter**	**Current Distributed** 3,560 kW	**Current Central** 118,000 kW
BoP CapEx	$257/kW	$118/kW
Mechanical BoP Cost	$136/kW	$36/kW
Electrical BoP Cost	$121/kW	$82/kW

Finally, the energy efficiency of the water electrolyzer is 70% (versus the LHV of hydrogen)[9,10] and the capital cost is 600 $/kWe.[9,10] The energy expenditure of pumps, compressors, and heat exchangers was calculated using thermodynamic equations.

For 99.99% purity, the pressure swing adsorption air separation unit can be modeled by equation (Equation 7):[11]

$$CapEx_{PSA} = 83903 * M_{N_2} + 29572 \tag{7}$$

The energy required for this system is 1420 kJ/kg_{N_2}.[11]

References

(1) Mongird, K.; Viswanathan, V. V.; Balducci, P. J.; Alam, M. J. E.; Fotedar, V.; Koritarov, V. S.; Hadjerioua, B. *Energy Storage Technology and Cost Characterization Report; PNNL-28866*; Pacific Northwest National Lab. (PNNL), Richland, WA, 2019; https://www.osti.gov/biblio/1573487 (accessed 2022-10-15).

(2) Nolte, C. High-resolution land value maps reveal underestimation of conservation costs in the United States. *Proc. Natl. Acad. Sci. U.S.A.* **2020**, *117 (47)*, 29577–29583.

(3) Andrews, S.; Smith, B.; Deceglie, M.; Horowitz, K.; Silverman, T. *National Renewable Energy Laboratory, Comparative PV LCOE Calculator, Version 2.0.0*; National Renewable Energy Laboratory, 2021; https://www.nrel.gov/pv/lcoe-calculator/ (accessed 2022-10-12).

(4) Here is how you can calculate the annual solar energy output of a photovoltaic system. `https://www.saurenergy.com/solar-energy-blog`, (accessed 2023-01-15).

(5) Smith, A.; Klosek, J. A review of air separation technologies and their integration with energy conversion processes. *Fuel Process. Technol.* **2001**, *70 (2)*, 115–134.

(6) Fernandez, C. A.; Hatzell, M. C. Editors' Choice—Economic Considerations for Low-Temperature Electrochemical Ammonia Production: Achieving Haber-Bosch Parity. *J. Electrochem. Soc.* **2020**, *167 (14)*, 143504.

(7) Tsuchiya, H.; Kobayashi, O. Mass production cost of PEM fuel cell by learning curve. *Int. J. Hydrog. Energy* **2004**, *29 (10)*, 985–990, Fuel Cells.

(8) Peterson, D.; Vickers, J.; DeSantis, D. *Hydrogen Production Cost From PEM Electrolysis - 2019*; U.S. Department of Energy DOE Hydrogen and Fuel Cells Program Record, 2020; https://www.hydrogen.energy.gov (accessed 2022-05-10).

(9) Schmidt, O.; Gambhir, A.; Staffell, I.; Hawkes, A.; Nelson, J.; Few, S. Future cost and performance of water electrolysis: An expert elicitation study. *Int. J. Hydrog. Energy* **2017**, *42 (52)*, 30470–30492.

(10) Buttler, A.; Spliethoff, H. Current status of water electrolysis for energy storage, grid balancing and sector coupling via power-to-gas and power-to-liquids: A review. *Renew. Sust. Energ. Rev.* **2018**, *82*, 2440–2454.

(11) Liu, Y.-H.; Fernández, C. A.; Varanasi, S. A.; Bui, N. N.; Song, L.; Hatzell, M. C. Prospects for Aerobic Photocatalytic Nitrogen Fixation. *ACS Energy Lett.* **2022**, *7 (1)*, 24–29.
